# Pharmacotherapy-Based Problems in the Management of Diabetes Mellitus: Needs Much More to be Done!

**DOI:** 10.4103/0975-1483.66801

**Published:** 2010

**Authors:** N Ali, SWA Shah, J Khan, S Rehman, M Imran, I Hussian, N Shehbaz, H Jamshed, S Khan

**Affiliations:** Department of Pharmacy, University of Malakand, Chakdara, Khyber Pakhtunkhwa, Pakistan; 1 Department of Pharmacy, Hayatabad Medical Complex Hospital, Peshawar, Khyber Pakhtunkhwa, Pakistan

**Keywords:** Diabetes mellitus, pharmacotherapy-based problems, ADRs, mismatches

## Abstract

A total of 856 diabetic patients were evaluated for pharmacotherapy-based problems like for possible drug interactions, adverse drug reactions, and other mismatches, if any. Poor correlation between the advised insulin therapy and patients’ fasting blood glucose levels (12%, n=103) was observed. To most of the patients (41.66%, n= 357), insulin therapy was advised in combination with glucocorticoides, thiazides diuretics, and propranolol. Prescribing beta blocker (propranolol) with insulin is contraindicated. The higher incidence of diabetic foot patients was in the mean age of 57±3.4 years that was controlled with combination therapy of insulin and oral antidiabetics (63.0%, n=516). 11.1% of the treated patients could not take the prescribed therapy due to poor acceptance of insulin therapy due to its syringe needle prick. 41.66% risks of potential drug interactions, 7.93% adverse drug reactions, and 6.6% mismatches were recorded, as per the international approved algorithm, for managing a diabetes mellitus that reflects poor health care system. All these events necessitate for coordinating with other health professionals to make the therapy safer in the better interest of the patients. It is concluded that in practice prescribing pattern carries more risks for patients. It is imperative to improve the practice of pharmacotherapeutics rather than to practice in routine.

## INTRODUCTION

Hyperglycemia may be due to insufficient insulin or poor response to insulin. Hyperglycemia may also be present with altered metabolism of lipids, carbohydrates, and proteins with increased risk of cardiovascular diseases.[1]

Sedentary life style is a major cause of obesity that is a factor for diabetes mellitus in middle-age population.[2] Disorder of lipid metabolism and hypertension may be seen in the diabetic patients.[3] It is an admitted fact that the larger the waist belt, the shorter will be the life and vice versa. If not treated, the hyperglycemia may go onto fulminant diabetes mellitus and its effects on end organ damage like kidneys, eyes, heart, and brain can be fatal in type II diabetes mellitus.[4,5] The prevalence of diabetes mellitus is attributed to ethnicity while living in the same environment.[6] However, western life style accelerates its prevalence. Diabetes mellitus can be better managed with control on diet, though pharmacotherapy is one the best option once the disease goes out of control.[7] However, poly-pharmacy carries risks of drug interactions and adverse drug reactions.[8] Hence, we analyzed retrospectively the treatment charts of the patients for possible pharmacotherapy therapy-based problems.

## MATERIALS AND METHODS

### Collection of data

Data were recorded, on the prescribed history form, using “purposive sampling techniques” over a period of 3 months in the respective teaching hospitals of the province. We could interview a total of 856 patients through skilled internees of Pharm-D Program in the prescribed period. Authenticity of the data was guaranteed as it was recorded in the prescribed history forms, concurrently, by the internees. The data were analyzed, retrospectively, for review of patients’ medication charts for possible pharmacotherapy problems.[9] Main drug therapy and supportive drug therapy with potential drug interactions are listed in Table 1.

**Table 1:** Main and supportive pharmacotherapy with potential drug interactions

Main regular therapy advised for the management diabetes mellitus			Potential drug interactions among the prescribed therapy and drug disease* interaction	
Characteristics	n	% of total†	Combination used	Remarks and interventions
All patient	856	100	INS + glucocorticoids	Increases blood sugar by stimulation of gluconeogenesis, mobilization of fats, decreases affinity of tissue receptors for insulin.[11] Take actions as necessary.
Insulin (INS) therapy‡	600	69.84	INS + thiazides	Thiazides cause hypokalemia that decreases insulin secretion and receptor sensitivity insulin.[12,13] Watch for diabetic control when thiazides are given together with any antidiabetic.
Oral hypoglycemics§	-	-		
Metformen (MTF)	640	39.68		
Glipazide	41	4.76	MTF + renal insufficiency*	Drug disease interaction as it increases the lactic acidosis that requires hemodialysis.[14]
Glibenclamide (GBD)	109	12.69		
Glimiperide (GLM)	81	9.52	INS + propranolol	Inhibition of insulin secretion and inhibition of the symptoms of hypoglycemia. Look for Cardio selective Beta blockers.[15]
Pregabaline (PRG)	68	7.93		
Gliclazide (GLD)	109	4.76	Aspirin + heparin	Avoid the combination as it may increase bleeding. Look for non salicyllates or give oral anticoagulant.[16]
Tolbutamide (TMD)	14	1.58	ACEIs + aspirin	Renal failure if combination used for months as supportive therapy Shift to acetaminophen instead aspirin.[17,18]

* Drug disease interaction

† Cumulative number may be more than 100% as more than one class of drugs was prescribed to same patient. Abbreviations are used in parenthesis

‡ Including type I and type II diabetic patients

§ Oral drugs prescribed to type II diabetic patients

Diabetes mellitus is some time associated with other concurrent ailments like diabetic foot (the major cause of hospitalization in diabetics); therefore, relatively more drugs were prescribed for the total management of type II diabetic patients. Hence, we considered the algorithm described by Cantrill and Wood,[7] and Barar[10,11] for the management of diabetes as standard protocols.

### Diagnosis and treatment

Respective ward physicians diagnosed the cases. All relative biochemical tests were performed as per the advice of respective ward physicians.

### Definitions

*Main drug therapy*: Drugs prescribed to control hyperglycemia that include either oral hypoglycemics or insulin therapy or combination of both as specified elsewhere in the text of this paper.

*Supportive drug therapy*: Drugs prescribed other than mentioned in the main drug therapy that mostly include antibiotic therapy, antiemetics, diuretics corticosteroids, and other drugs for concurrent ailments either to increase the potency or to decrease the side effects, if any.

*Adverse drug reaction*: An adverse drug reaction was defined as an unwanted effect associated with drug therapy, including main and supportive drug therapies that could lead to either discontinuation or altering the course of therapy.

*Potential risks of drug interactions*: Combination of two or more than two drugs used in the therapy with reported harmful effects on the health or quality of life of the recipients that is/are reported in the medical literature that necessitates for either change in the dose regimen or to search for suitable therapeutic alternatives.

### Statistical analysis

Microsoft XL sheet was used to calculate the percentage, mean, and standard deviation for variables mentioned elsewhere in the text.

## RESULTS AND DISCUSSION

As mentioned in Table 1, total 856 diabetic patients were admitted in the hospitals. Therapy was advised for control of their major illness i.e. hyperglycemia with concurrent cases like diabetic foot. It is evident that to 69.84% cases (n= 600, type I and type II inclusive), insulin therapy was advised. In certain cases, combination therapy of insulin and oral hypoglycemics (60.3 %, n= 516) were tried to control hyperglycemia. According to Table 1, among the prescribed oral hypoglycemics, metformen was most frequently (39.68 %, n= 360) prescribed. The oral hypoglycemics were of the order: metformen (39.68%) > glibenclamide (12.69%) > glimeperide (9.52 %) > pregabaline (7.93%) > gilipizide (4.76%) = gliclazide (4.76%) > tolbutamide (1.58%). Interestingly, the hyperglycemia was controlled with insulin therapy. But there was no correlation of blood glucose levels with insulin therapy that lead to hypoglycemia that was observed in 12% of the reported cases (data not shown). The main reason of negligence, in this regard, was due to the fact that the laboratory reports were received at 11:00 am and the patients had to receive their advised insulin therapy in the morning between 8:00 and 9:00 am that reflects poor correlation of the therapy with patients’ biochemical tests. In Table 1, as per reported medical literature, potential drug interactions are listed that required interventions but were not intervened. To most of the patients (41.66%, n= 357), insulin therapy was advised in combination with glucocorticoides, thiazides diuretics, and propranolol. Insulin and glucocorticoids combination increases hyperglycemia and decreases affinity of tissue receptors response to insulin.[11,12] It is noteworthy that thiazide diuretics decreases the sensitivity of insulin to its receptors and of course its secretion as well.[13,14] Metformen was prescribed to a renally impaired patient (a contrindications of metformen).[15] Combination of beta blockers (BBs) can be dangerous not only in a sense that BBs decrease the secretions of insulin from the islets of Langerhans but also mask the symptoms of reflex tachycardia that is regarded as a vital force (to me personally) in combating hypoglycemia and can be fatal.[16] Combination of aspirin and heparin combination have reported with GIT bleeding[17] that was not intervened as suggested in Table 1. The higher incidence of diabetic foot patients was in the mean age of 57±3.4 years (data not shown). Hence, more care should be exercised in patients with mean age 57±3.4 years and insulin regimen should be incorporated for the management of hyperglycemia to help prevent them from complication of diabetic foot rather than to wait and let the patient goes on to complications.[19,20] In this regard, a comprehensive awareness program is necessary to change the mind set of diabetic patients about the insulin therapy and give them awareness about diabetic foot as a complication. Having done this accordingly, as stated earlier, the patients will keep on taking advised insulin therapy and they will not go onto diabetic foot, the major cause of hospitalization.[19,20] All the events related to pharmacotherapy problems are summarized in Table 2 in light of the algorithm described by Yarborough PC.[9] Out of the total patients, 11.11% could not take the prescribed drugs due to pharmaceuticalbased problems like poor acceptance to insulin syringe prick. The reported ADRs were diarrhea (7.69%), burning micturation, and weakness (2.0%). 41.66% of the cases carried potential risks of drug interactions and the most frequently encountered drug interactions are mentioned in Table 1. Self-explanatory mismatches are summarized in Table 2 that are 20.0% in the main drug therapy and 41.26% in the supportive drug therapy that lead to a high antibiotic number per patients (2.4/patient). To 6.6% of the cases, no relevant drug therapy was prescribed for indications such as hypertension, hepatitis, diarrhea, fatigue, and burning feet syndrome and stress. This reflects poor patient’s health care system that requires improvements in light of the practice of rational therapeutics. Hence, it is imperative to improve the practices of therapeutics by providing our health professionals a patient’s based learning rather than to practice in routine.[21] Poly-pharmacy leads to many ADRs that can be anticipated by interdisciplinary integration of disciplines such as medicine, pharmacy, and other health professions for the promotion of practice of rational therapeutics. A “medication review team”[22] including pharmacists and physicians may be constituted throughout the country to give concise recommendations and corrective measures for proper utilization of drugs.

**Table 2:** Data extracted from the history forms to show the % incidence of pharmacotherapy based problems*

Pharmaceutical based problems		Risks to patients		Mismatches				Efficacy
				Main drug therapy Supportive drug therapy				
Patients not receiving prescribed drugs	Routine monitoring missing like correlation with lab findings	Adverse Drug Reactions	Drug Interactions	No indications for current drug prescribed	Indication for drug exists but not prescribed	No indications for current drug prescribed	Indication for drug exists but not prescribed	Wrong drug or regimen prescribed / more efficacious choice available
11.11%	12%	7.93%	41.66%	20.6%	0%	41.26%	6.6%	4.7%

* %Cumulative incidence may be more than 100 as more than one type of problems were observed in same patient.
